# An Enduring Shell Artefact Tradition from Timor-Leste: *Oliva* Bead Production from the Pleistocene to Late Holocene at Jerimalai, Lene Hara, and Matja Kuru 1 and 2

**DOI:** 10.1371/journal.pone.0161071

**Published:** 2016-08-18

**Authors:** Michelle C. Langley, Sue O‘Connor

**Affiliations:** Archaeology & Natural History, School of Culture History & Language, College of Asia & the Pacific, Australian National University, Canberra, Australia; Universidade do Algarve, PORTUGAL

## Abstract

In this paper, we describe 485 *Oliva* spp. shell beads recovered from four archaeological cave sites Jerimalai, Lene Hara, Matja Kuru 1, and Matja Kuru 2, located in Timor-Leste, Island Southeast Asia. While Pleistocene-aged examples of modified marine shells used for personal ornamentation are common in African and Eurasian assemblages, they are exceedingly rare in Southeast Asia, leading some researchers to suggest that these Modern Human societies were less complex than those found further west. In Timor-Leste, the lowest *Oliva* bead to be recovered was directly dated to ca. 37,000 cal. BP, making it the oldest piece of personal ornamentation in Southeast Asia. Morphometric, taphonomic, use wear, and residue analyses of these beads alongside modern reference specimens, and experimentally made examples indicate that the *Oliva* shells were modified to be strung consecutively (as in a necklace), and while their mode of production changed remarkably little over the thousands of years they were utilised, an increase in their deposition around 6,000 cal. BP suggests that there was a change in their use coinciding with sea-level stabilisation. These tiny beads demonstrate that early Island Southeast Asian societies produced the same kinds of symbolic material culture we have come to expect from the more intensively studied African/Eurasian region, and that limited sampling and poor recovery methods have biased our perspectives of this region.

## Introduction

Representing a behaviour specific to humans whereby standardised items are displayed on the physical body to project symbolic meaning to members of the same or nearby groups, ornamentation in the form of beads or pendants made from marine shells and animal teeth, continues to be one of the best sources of information regarding social behaviour in early Modern Human communities (e.g., [1–11]). Until recently, the vast majority of early instances of shell bead use were reported from Eurasian and African contexts (e.g., [2, 4–5, 9, 12–13]), with Greater Australian examples of Pleistocene antiquity rare [14–16], and Southeast Asian one’s even more so. Indeed, while there have been several significant finds of perforated animal teeth and shells in north and central Chinese sites such as Zhoukoudian, Xiaogushan, and Shuidonggou 2 [17–18], to date the only convincing examples of Pleistocene-aged pieces of personal adornment from Southeast Asia consist of two perforated mammalian teeth from Xom Trai Cave and Du Sang Rockshelter, Vietnam dated to between 22,000 and 19,000 cal. BP [19].

This paucity of remnants of past symbolic behaviour has led some researchers to posit that the earliest Modern Human communities in Southeast Asia were materially less complex than those located in Africa and Europe. It has even been suggested that as biologically and behaviourally Modern Human populations moved away from their African homeland, they somehow ‘lost’ key technological features which separated them from more ‘archaic’ hominid groups [20]. While such arguments have largely been centred on lithic technology, the lack of personal ornamentation throughout Southeast Asia has supported such notions. Additionally, early excavations throughout this region—while large—were poorly controlled by modern standards with their deposits sieved through very coarse mesh screens, if sieved at all. Consequently, had beads been present in any of these explored deposits they would not have been collected, while those recovered from more recently excavated pre-ceramic layers have been assumed to have been displaced from higher Neolithic levels. With no direct dating of individual beads, these finds have gone largely unstudied and unintegrated into a more concise understanding of human adornment in Southeast Asia.

Significantly, recent excavations in Timor-Leste, Island Southeast Asia (ISEA), have unearthed numerous marine shell beads simply through utilising fine meshed sieves. The controlled excavation and recovery techniques, coupled with a methodical dating regime, including the direct dating of individual pieces of shell material culture, have resulted in the identification of early instances of shell fishhooks [21], and shell beads [22–24]. Here we report an assemblage of 485 *Oliva* spp. shell beads recovered from Jerimalai, Lene Hara, Matja Kuru 1 (MK1), and Matja Kuru 2 (MK2) in Timor-Leste. Found throughout the deposits of these four sites, the oldest example was directly dated to 38,246–36,136 cal. BP, while the most recent beads are only a couple of hundred years old. This collection demonstrates that early ISEA societies produced the same kinds of personal ornamentation as the more intensively studied African/Eurasian region, and that limited sampling and poor recovery methods have biased our perspectives of a region which in actuality houses a rich record of symbolic material culture.

## Archaeological Context

**Fig 1:**
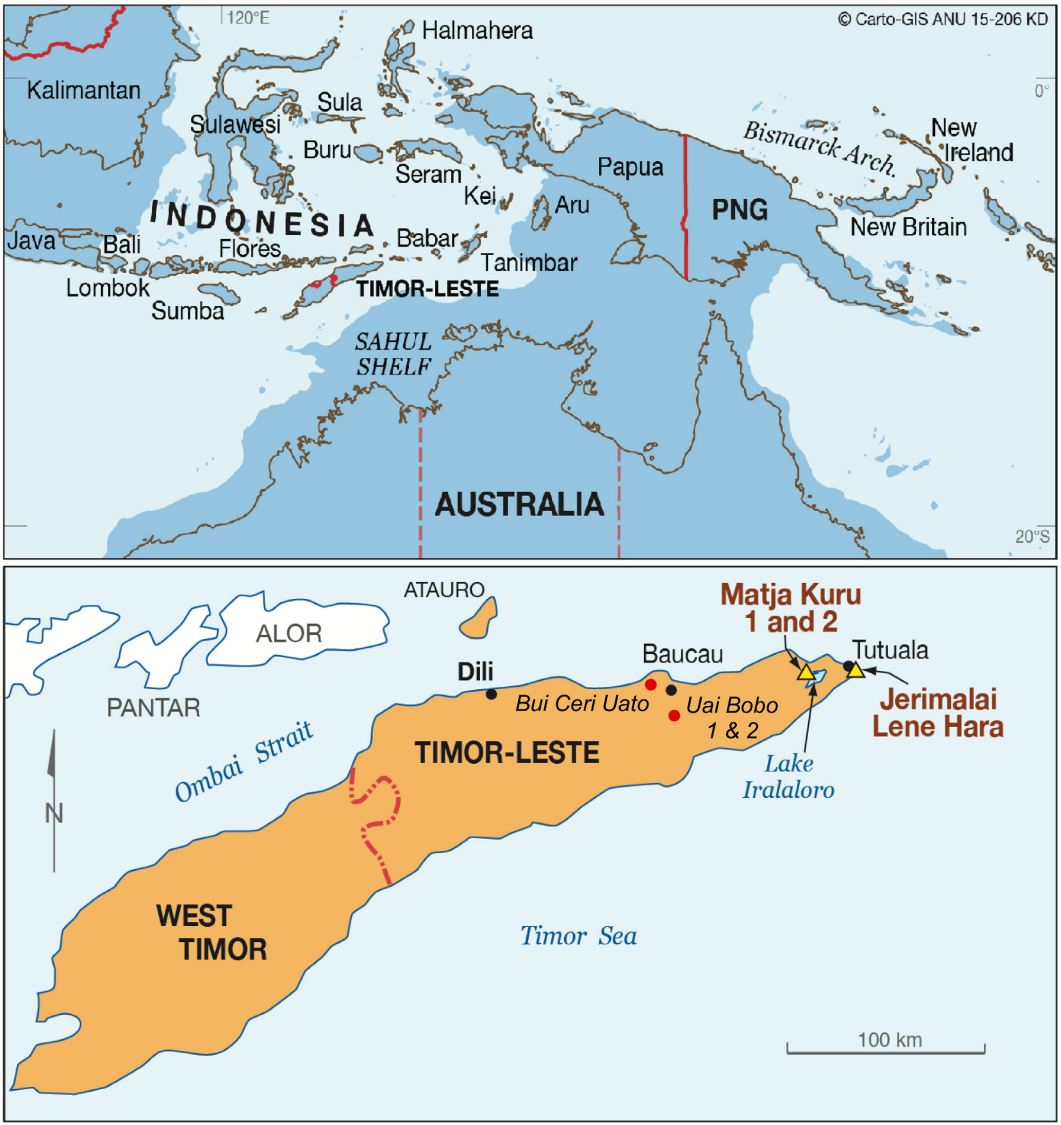
Location of Jerimalai, Lene Hara, Matja Kuru 1, and Matja Kuru 2. Printed with permission from CAP CartoGIS, College of Asia and the Pacific, ANU, original copyright, 2015.

The sites of Jerimalai (8'24.84' S, 127'17.50' E) and Lene Hara (8' 24.35' S, 127'17.58' E) are located less than a kilometre apart and within a kilometre of the current coastline at the easternmost tip of Timor-Leste, southeast of the village of Tutuala (Fig 1). They are formed in uplifted Pleistocene marine terraces which run parallel to the coastline. Jerimalai is a coralline limestone shelter approximately 75 m above present sea level, while Lene Hara sits at 100 m above present sea level (based on GPS using WGS 84).

Excavation of Jerimalai was undertaken during July 2005, when two 1 x 1 m square test pits were dug in the eastern and central area of the shelter (Squares A and B). The Jerimalai deposit has an upper ceramic-rich and a lower aceramic horizon, and was excavated in approximate 1–5 cm (average depth of 2.2 cm) spits within stratigraphic units, where such could be identified. Wet screening of all cultural deposits through 1.5 mm mesh ensured recovery of even very small finds, including lithic micro-debitage and shell beads. Pottery occurred in the upper horizon, with stone artefacts, shell, and bone remains being found throughout the deposit. Owing to the fact that more than 50 kg of anthropogenic marine shell was excavated from these two squares, 3-D piece plotting of individual shell artefacts was not feasible, though finished beads and obviously worked pieces of shell were separated and individually bagged when identified during wet sieving and sorting in the field. Other pieces were only identified once back in the laboratory.

**Fig 2:**
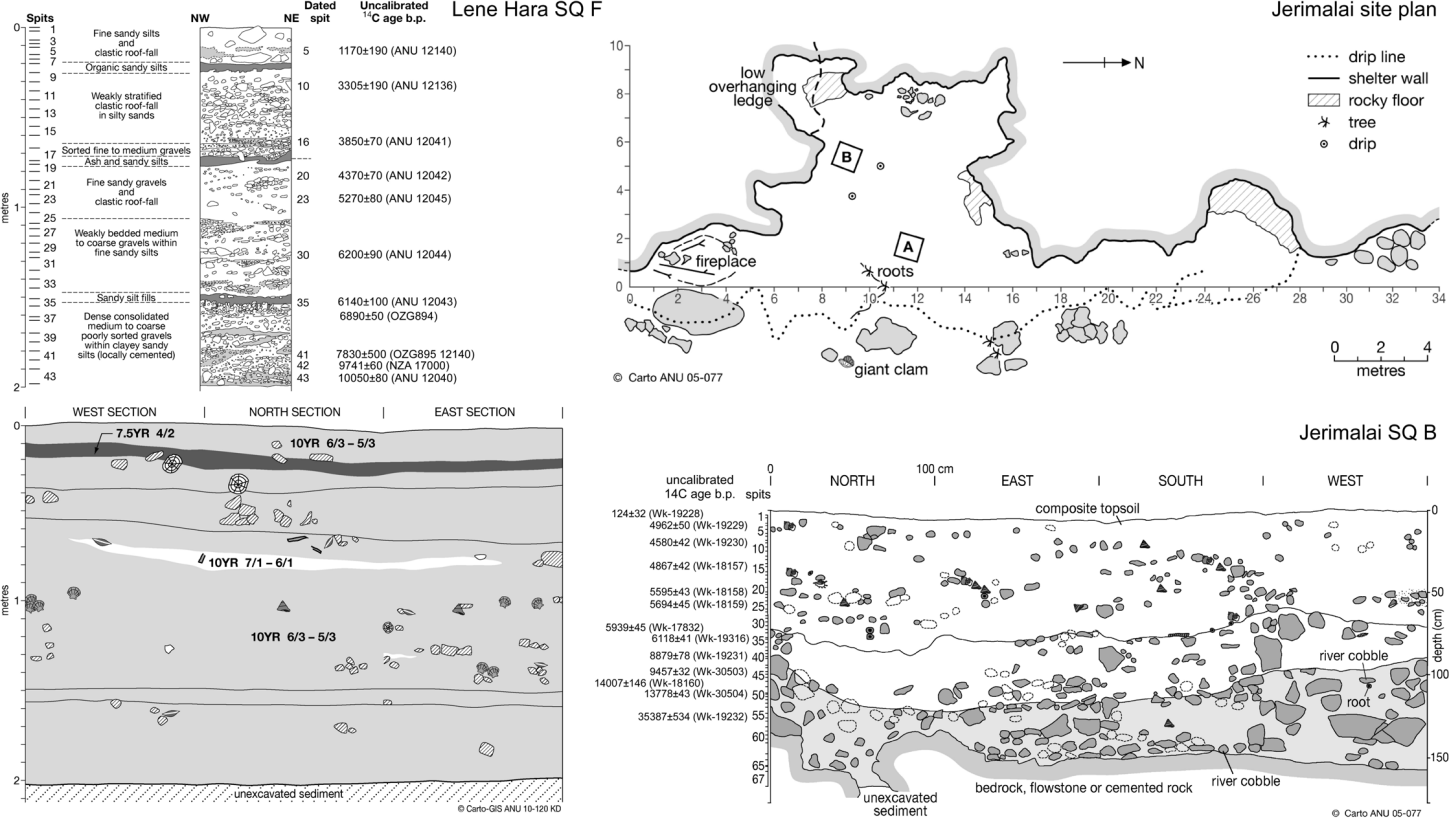
Stratigraphic sections of Lene Hara Square F and Jerimalai Square B. Printed with permission from CAP CartoGIS, College of Asia and the Pacific, ANU, original copyright, 2015.

While the analysis of the Jerimalai cultural materials is ongoing, radiometric dating of the lower levels has already demonstrated that it is the oldest modern human occupation site in ISEA east of the Sunda Shelf [21, 25]. The lowest sample from Test Pit A is dated to 43,381–41,616 cal. BP (38,255±596 BP—Wk-17831), while Test Pit B returned a date of 42,475–41,125 cal. BP (37,267±453 BP—Wk-17833) (Fig 2). These samples place first occupation at Jerimalai at before 42,000 cal. BP. The radiocarbon dates indicate that the site saw little habitation during the Last Glacial Maximum. The sites was occupied in the early Holocene seeing more intense occupation after about 6000 cal BP as sea level came close to its present position. While the currently available dates suggest that some depositional mixing has occurred in the middle section of the Jerimalai sequence (in levels dated to between c. 9,000 and 16,000 cal. BP; Table 1), we believe that post-depositional disturbance of these, and the lower levels, is minimal for two reasons: (1) some of the dates were obtained on shell artefacts, particularly *Oliva* shell beads which may represent heirloomed or reused artefacts deposited long after their creation (discussed below); and (2) the deposit becomes increasingly compact and carbonate cemented at depth, reducing the amount of bioturbation and other post-depositional disturbances that may affect the oldest layers.

**Table 1:** Distribution of worked Oliva spp. shell in Jerimalai, Square A.

SPIT	DATE	Material Dated	C14 2ơ cal yr B.P.	Finished Beads	Ochre Present
1				4	2
2				3	1
3	2570±34 (Wk-19224)	Charcoal	2749–2485	2	1
4				3	0
5	3245±39 (Wk-19225)	Charcoal	3560–3348	3	2
6	5341±41 (Wk-18154)	*Turbo* sp.	5835–5598	5	2
7				12	9
8				2	1
9				14	10
10				4	2
11				8	5
12	5567±44 (Wk-18155)	*Turbo* sp.	6100–5866	8	6
13	5549±62 (Wk-17829)	*Turbo* sp.	6115–5762	9	7
14				4	4
15				3	1
16				2	1
17				2	1
19				1	0
21	5909±40 (Wk-19226)	*Turbo* sp.	6422–6245	1	0
22				2	0
23				1	1
26	10,110±79 (Wk-18156)	*Haliotis* cf. *varia*	11,245–10,870		
27	19,952±235 (Wk-17830)	*Turbo* sp.	23,935–22,564	2	1
28				2	2
29				1	1
30				2	1
31				1	0
32				1	1
38	13,658±91 (Wk-19227)	*Turbo* sp.	16,761–15,455		
42				2	1
44	7549±27 (ANU-48107)	*Oliva* shell bead	8096–7934	1	1
46	38,255±596 (Wk-17831)	*Trochus*	43,381–41,616		
				**105**	**64**

At Lene Hara, Test Pit F—the 1 x 1 m square from which all of the Lene Hara beads reported herein originated—was excavated in the lower northern chamber during 2002 [26–28]. This pit was dug to a depth of 2.05 m without reaching bedrock, with pottery recovered down to 0.7–0.75 m below the surface—this transition from ceramic to aceramic deposits clearly defined (Fig 2). The lowest level of the pit was dated to 35,192–33,896 cal. BP (30,950±360 ANU-11401). Preservation of both shell and bone is broadly similar at all excavated levels, and as with other parts of the site, charcoal was not found below the upper 0.2 m of the deposit. Thus, marine shell was used for dating in Test Pit F. The radiocarbon dates support a model of net accumulation and continuous deposition throughout the Holocene in this northern section of the cave. It was from this square that the early Holocene fish hook was recovered from Spit 42, which is dated to c. 10,500 cal. BP (9741±60 NZA-17000) [26].

The cave sites of MK1 (8'24.87' S, 127'07.36' E) and MK2 (8'24.88' S, 127'07.42' E) are located adjacent to each other in an uplifted limestone ridge north east of the modern village of Poros, and only a few hundred meters north from Lake Ira Lalaro. They are approximately 370 m above sea level and around 8 km in straight-line distance from the north coast of Timor-Leste (Fig 1), with MK2 a few hundred meters to the east of MK1 along the same cliff line. Archaeological investigation of these sites consisted of a 1 x 2 m test-pit (conjoined Squares A and AA) excavated at MK1, while a 1 x 1 m test-pit was sunk into MK2 (Square D).

At MK1, the oldest date returned was 16,355–15,566 cal. BP (13,690±130 ANU-11616 from Square AA, Spit 21), though most of the deposit built up between 5600–4600 BP [29]. Archaeological material consists of stone artefacts along with lake fauna such as freshwater turtles and fish, terrestrial faunal such as large rodents, as well as marine shellfish and fish, with these last being a continuous presence throughout the sequence. Some disturbance of the MK1 lower deposit is indicated by an inversion in dates—a date of 11,098–10716 cal. BP (9940±60 OZF-784) was obtained from a unit 15–20 cm lower in the profile (Spit 25) than a date of 16,355–15,566 (13,690±130 ANU-11616) from Spit 21.

Human use of MK2 begins sometime prior to 35,000 years ago with an age estimate of 36,866–35,285 cal. BP (32,220±300 OZF-785) for a near basal unit (Spit 47) returned, though it should be noted that excavation was discontinued prior to reaching bedrock [30]. Dating of Spits 41 and 44 at MK2 confirmed this antiquity, with ages of 36,268–34,649 cal. BP (31,060±310 NZA-16177) and 35,882–34,575 cal. BP (31,660±320 NZA-16178) respectively. At this site, cultural material includes stone artefacts, faunal remains, and marine shellfish which were most abundant in the lowest levels dated to between c. 32,000 BP and 31,000 (Spits 49–41) [29]. A stone-lined oven dated to around 10,000 years BP was uncovered in Spits 19–25, while a dog burial dug from higher up was found in Spit 25 and directly dated to 2921–3075 cal. BP (2967±50 WK-10051) [31].

Both MK1 and MK2 appear to have been used largely simultaneously. MK2 constituted an attractive temporary campsite around c. 30,000 years ago, before abandonment during the Last Glacial Maximum (LGM) and then occasional reoccupation across the Pleistocene-Holocene transition. This site then appears to have become much less attractive to people, perhaps owing to an opening of a hole in the roof at the back of the shelter [30]. MK1 was not used until 15,000 years ago, when unlike MK2, it still provided good shelter and continued use of the space resulted in sedimentary buildup increasingly creating a conveniently flat surface within the shelter. Most intense use of both sites occurred during the mid-Holocene period [30].

## Methods

The identification of the *Oliva* shells as humanly modified items of adornment was based on the following: (1) evidence for human agency in their selection, transport, and accumulation at each of the four sites; and (2) the presence of clearly identifiable manufacturing marks and use wear demonstrably distinct from taphonomic traces. In order to differentiate between these natural and anthropogenic factors, two lines of investigation were followed: (1) the examination of low-tide collected live and dead specimens of *Oliva* spp., and (2) the experimental creation of *Oliva* shell beads. Review of published criteria for identifying anthropogenic alterations of shell beads, such as that provided in d’Errico et al. [4], Vanhaeren et al. [10], Stiner et al. [32], and Cristiani et al. [33], supplemented these primary modes of investigation.

Reference collection examples which were examined as part of this study included more than 80 newly gathered *Oliva oliva* (a larger but morphologically and behaviourally similar species to the *Oliva brettinghami* which compose the archaeological assemblage) from Kawara Beach in north Queensland. This non-endangered species is abundant throughout the estuarine zone surrounding Kawara Beach, where collecting is permitted without need for permits from the Queensland government. These shells were gathered over several visits to the area over the course of two months. Additionally, a range of *Oliva* spp. specimens curated in the Archaeology and Natural History Osteology Reference collection held at the Australian National University were included in the study. These shells were examined using a Zeiss 2000-C stereo microscope fitted with an AxioCam MRc5 camera in order to identify the types, intensity, distribution, and frequency of natural damage present on beach collected specimens.

**Fig 3:**
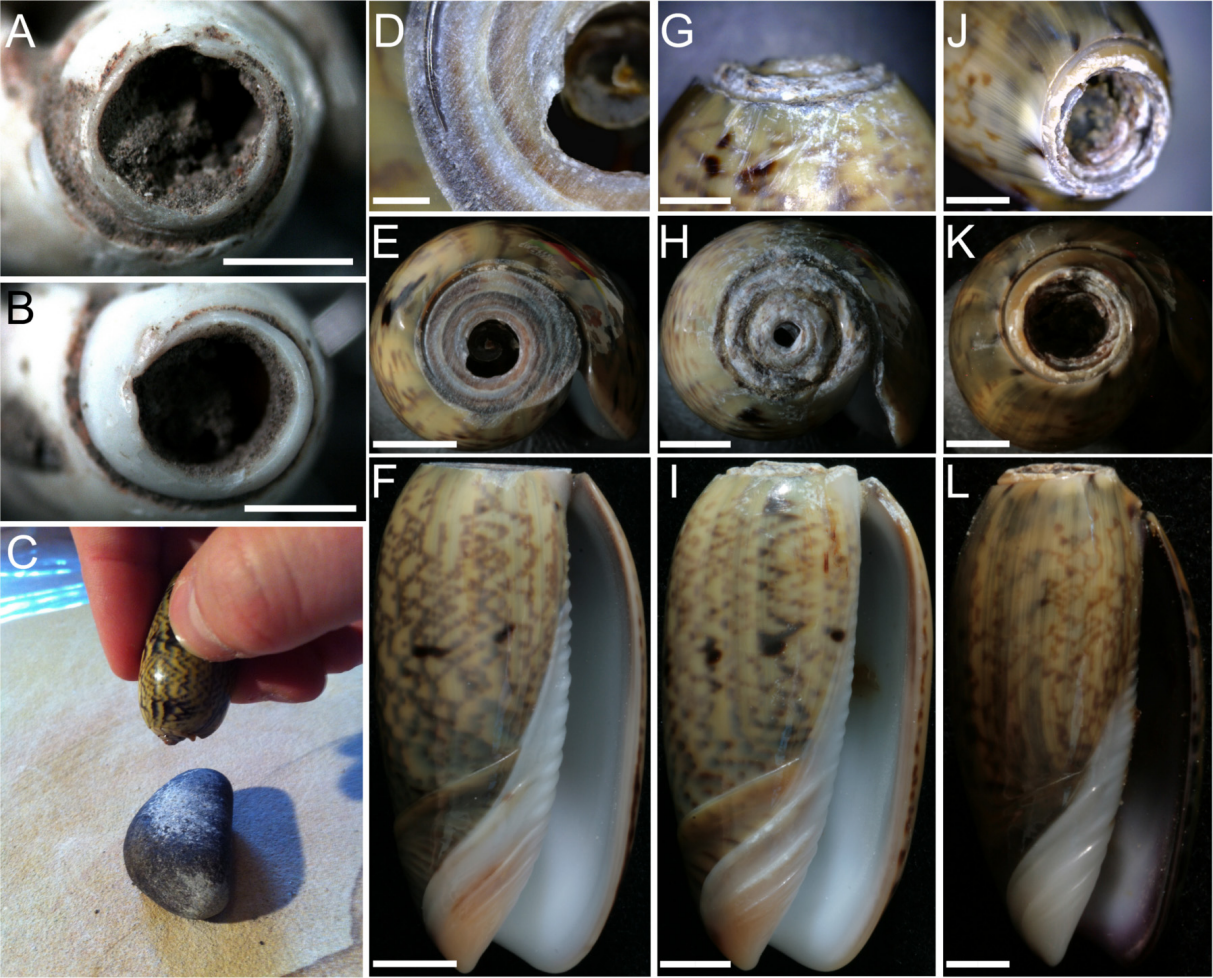
*Oliva oliva* shells experimentally perforated. (D—F) grinding on a sandstone slab; (G—I) percussion flaking where the shell is held stationary and a stone is brought down against the apex; and (J—L) percussion flaking where the shell apex is repeatedly struck on a hard surface—the ‘tapping-technique’ (C). (A and B) Jerimalai artefacts. Bar scale = 1 millimetre.

**Fig 4:**
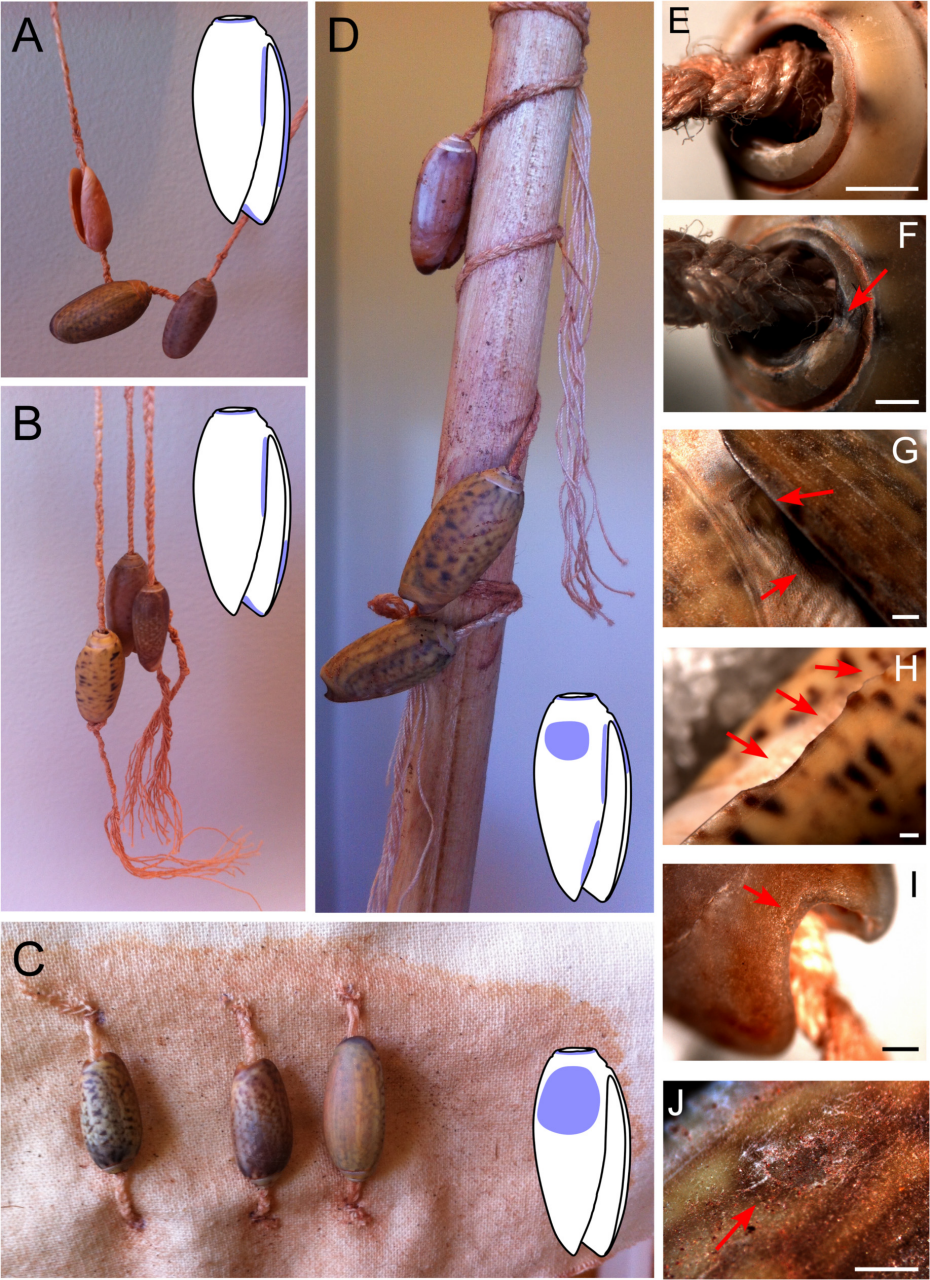
Experimental stringing of *Oliva oliva* shells. (A) consecutive stringing—C1; (B) grouped singularly strung beads—C2; (C) singularly strung beads attached to cotton canvas—C3; and (D) strung beads attached to a dowel rod—C4. Accumulated wear after 3 hours of shaking: (E—F) smoothing of aperture on a C1 (E) and C2 (F) beads; (G) developing facet on the inner lip of a C1 bead; (H) developing facet on the outer lip of a C2 bead: (I) developing facet on the canal of a C2 bead; (J) wear of the body whorl on a C3 bead. Bar scale = 1 millimetre.

Next, a series of experiments was undertaken in order to create *Oliva* shell beads bearing anthropogenic alterations of known origin in terms of manufacture method and use type. Three alternative methods of apex perforation were explored using 15 of the *Oliva oliva* shells collected from Kawara Beach: (1) grinding on a sandstone slab; (2) percussion flaking, where the shell is held stationary and a stone is brought down against the apex; and (3) tapping, where the shell apex is forcibly tapped repeatedly on a hard surface (Fig 3). Next, 12 of these experimental beads (those most similar in size) were selected for stringing experiments. Twelve strands of pure cotton thread were plaited together to create a cord approximately 1.5 mm in width, and three beads were strung into four differing configurations (shown in Fig 4) based on review of the ethnographic use of *Oliva* shells. Following Vanhaeren et al. [10], the shells were shaken for three hours stopping every 15 minutes to soak both the shells and their threads in a mixture of water, vinegar, and red ochre. This mixture of water, vinegar, and red ochre was used to simulate the grit, sweat, and ochre (this last indicated by residue analysis) the shell beads would have come into contact with when worn on the human body (after Vanhaeren et al. [10]). Each of the shells was then inspected for signs of wear using magnifications between 65–160 x with the same Zeiss 2000-C stereo microscope mentioned above. These experiments enabled the positive identification of modifications resulting from anthropogenic actions and their differentiation from similar appearing natural alterations.

At the completion of the experiments, each of the archaeological Timorese *Oliva* beads was individually examined using the same microscope set up, with anthropogenic traces mapped onto drawings of each shell, these drawings produced from high-resolution digital photographs of their dorsal and ventral surfaces and the use of an illustrating programme (*Canvas XII*). During this process, a red residue was observed on more than half of the shells (Tables 1–6), and consequently, two of the Jerimalai beads with dense concentrations of the residue (from SQ A Spits 2 and 7) were subjected to Laser Ablation ICP-MS (LA-ICP-MS) analysis in order to determine whether their chemical composition was consistent with ochre. As the observed red residue is uniform in its appearance and consistency across the entire Timor collection, it was felt that testing more of the beads was unnecessary.

**Table 2:** Distribution of worked Oliva spp. shell in Jerimalai, Square B.

Spit	Date	Material Dated	C14 2ơ cal yr B.P.	Finished Beads	Ochre Present
2				1	0
3	124±32 (Wk-19228)	charcoal	10–273	2	0
4	4962±50 (Wk-19229)	*Turbo*	5454–5131	3	0
5				7	3
6				4	1
7				3	1
9	4580±42 (Wk-19230)	*Trochus*	4900–4640	1	0
10				10	5
11				2	1
12				1	1
13				3	3
14				5	5
15				6	2
16	4867±42 (Wk-18157)	*Trochus*	5293–5032	1	0
18				9	5
19				9	5
20				6	5
21	5595±43 (Wk-18158)	*Trochus*	6123–5887	7	5
22				6	3
23	5694±45 (Wk-18159)	*Trochus*	6232–5972	16	12
24				12	11
25				2	1
26				7	2
27				9	4
28				7	5
29				5	4
30				8	4
31				8	5
32				5	4
33	5939±45 (Wk-17832)	*Trochus*	6455–6265	3	2
34	6118±41 (Wk-19316)	*Nautilus* shell bead	6654–6434	2	1
35				5	3
37				4	1
38				1	1
39				4	2
40	8879±78 (Wk-19231)	*Trochus*	9776–9393	1	0
41	6223±26 (Wk-30500)	*Oliva* shell bead	6755–6586	1	na
42	5575±27 (Wk-30501)	*Oliva* shell bead	6057–5887	1	na
43	13,901±45 (Wk-30502)	*Oliva* shell bead	16,461–16,057	1	na
46	9457±32 (Wk-30503)	*Oliva* shell bead	10,406–10,207	1	na
49	14,007±146 (Wk-18160)	*Haliotis* cf. *varia*	17,083–16,151		
50	13,778±43 (Wk-30504)	*Haliotis* cf. *varia*	16,261–15,907		
56	• 33294±380 (ANU-48106)	• *Oliva* shell bead	• 38,246–36,136	1	0
	• 35,387±534 (Wk-19232)	• *Turbo*	• 41,217–38,853		
				**190**	**107**

**Table 3:** Distribution of worked Oliva spp. shell in Matja Kuru 1, Square A.

SPIT	DATE	Material Dated	C14 2ơ cal yr B.P.	Finished Beads	Ochre Present
1				2	1
5	4650±70 (ANU-11835)	*Lambis lambis*	5115–4680		
8	5005±40 (NZA-16135)	*Lambis lambis*	5456–5274	1	0
10				1	0
11				1	0
12	3776±40 (NZA-17007)	*Chiton* sp.	3832–3595		
14	3840±70 (ANU-11632)	*Cymbiola vespertilio*	3985–3597	3	2
15				3	0
16				2	0
17				2	1
18				1	1
20				1	1
21				2	1
22				1	0
23				2	0
24				2	0
25	5720±50 (OZF-782)	*Trochus* sp.	6256–5992	3	2
27	5280±80 (ANU-11624)	*Turbo chrysostomus*	5848–5475	2	0
30				2	2
31	5680±110 (ANU-11623)	*Trochus niloticus*	6317–5851		
33				1	1
				**32**	**12**

**Table 4:** Distribution of worked Oliva spp. shell in Matja Kuru 1, Square AA.

SPIT	DATE	Material Dated	C14 2ơ cal yr B.P.	Finished Beads	Ochre Present
2				2	0
3	4420±70 (ANU-11834)	*Lambi lambis*	4792–4413		
5	4770±110 (ANU-11622)	*Turbo chrysostomus*	5323–4784	1	1
6				2	1
7	5620±70 (ANU-11621)	*Strombus luhuanus*	6193–5910	1	1
8				1	1
9	5010±60 (ANU-11620)	*Conus* sp.	5537–5238	4	3
10				7	5
11	4640±70 (ANU-11619)	*Lambi lambis*	5069–4641	3	3
12				5	4
13				5	1
14	5240±70 (ANU-11618)	*Trochus niloticus*	5767–5442		
15				2	2
16				2	2
17				1	1
18	5060±80 (ANU-11617)	*Turbo chrysostomus*	5585–5255	3	2
19				1	1
20	5090±100 (ANU-11789)	*Turbo argyrostomus*	5665–5224	6	4
21	13690±130 (ANU-11616)	*Strombus luhuanus*	16,355–15,566	1	0
22				4	3
25	9940±60 (OZF-784)	*Trochus* sp.	11,098–10,716	2	1
				**51**	**39**

**Table 5:** Distribution of worked Oliva spp. shell in Matja Kuru 2, Square D.

SPIT	DATE	Material Dated	C14 2ơ cal yr B.P.	Finished Beads	Ochre Present
10	2450±40 (NZA-16136)	*Modulus philippinosus*	2255–1978		
13	• 2510±50 (OZG-537)	•*Celtis sp*.	• 2717–2364		
	• 3190±40 (OZG-538)	•*Marine shell*	• 3455–3239		
15	8966±55 (NZA-18656)	*Nautilus* shell bead	9817–9499	1	1
16				1	1
17	10292±60 (NZA-17008)	*Chiton* sp.		1	1
22					
24	4490±40 (OZG-896)	Drilled *Trochus* shell bead	4808–4554	1	1
25	10078±60 (NZA-17009)	*Chiton* sp.	11,215–10,884		
26	9650±55 (NZA-16137)	*Chiton* sp.	10,691–10,381		
27				2	2
29				1	0
31	9190±50 (OZG-899)	*Nautilus* shell bead	10,200–9909		
32	• 9205±55 (NZA-17001) • 9260±60 (OZG-897)	• *Nautilus* shell bead • *Oliva* Shell bead	• 10,172–9813 • 10,215–9890	1	
35	9260±50 (OZG-898)	*Nautilus* shell bead	10,200–9909		
				**8**	**6**

**Table 6:** Distribution of worked Oliva spp. shell in Lene Hara, Test Pit F.

SPIT	DATE	Material Dated	C14 2ơ cal yr B.P.	Finished Beads	Ochre Present
?				1	1
7				1	1
10	3305±190 (ANU-12136)	*Trochus niloticus*	3603–2721		
15				1	1
16	3850±70 (ANU-12041)	*Trochus niloticus*	4003–3607	6	5
17				2	2
20	4370±70 (ANU-12042	*Trochus niloticus*	4775–4345	2	1
21				5	4
22				5	4
23	• 4900±40 (OZG-893)	• *Nautilus shell bead*	• 5313–5051	8	6
	• 5270±80 (ANU-12045)	• *Trochus niloticus*	• 5841–5466		
24				11	10
25				12	9
26				24	23
27	5782±45 (NZA-16998)	*Nautilus shell bead*	6300–6085	4	4
28				4	4
29				1	1
30				2	2
31				2	2
33				1	0
35	6890±50 (OZG-894)	*T*. *niloticus fish hook*	7500–7295	1	1
36				1	0
37				1	0
40	7945±65 (NZA-16999)	*Oliva shell bead*	8556–8283	3	2
41	7830±50 (OZG-895)	*Oliva shell bead*	9575–7378	1	na
42	9741±60 (NZA-17000)	*T*. *niloticus fish hook*	10,841–10,491		
43	10050±80 (ANU-12040)	*Tridacna maxima*	11,209–10,791		
				**99**	**83**

Finally, nine of the *Oliva* shell beads were directly dated using the AMS method to more firmly place these small pieces of ornamentation into the Timorese chronology (Tables 1–6). These dates, along with all others presented in this paper are given as calibrated (cal. BP), unless citing those published by other authors. Dates were calibrated in OxCal v.4.2 [34]. Charcoal radiocarbon samples were calibrated using the SHCal13 Southern Hemisphere curve [35], and marine shell samples calibrated using the Marine13 modelled ocean average curve [36]. No ΔR values were used in the calibration of marine samples owing to the unknown values for the region. A local ΔR has not been determined for Timor-Leste, but it is likely to be small with values throughout Indonesia and along the northern coast of Australia averaging 32 ± 41 ^14^C years [37–41]. All dates are given to 95.4% probability.

The shell bead collections of Jerimalai, Lene Hara, MK1, and MK2 are currently housed in the laboratories of Archaeology & Natural History, School of Culture, History & Language, College of Asia & the Pacific, at the Australian National University in Canberra, Australia and will be returned to Timor-Leste at the completed of the Laureate project. Their final place of depositary and future access will be determined at that time by our Timorese counterparts.

## Materials

The Timor *Oliva* bead assemblages are predominantly composed of *Oliva brettinghami*, a common species in the Indo-Pacific region [42], though other, rarer *Oliva* shells are also present (namely *Oliva textilina*, *and Oliva ceramensis*). *Oliva brettinghami* (cf. *Oliva caldania*) is a small species of Olive shell, typically reaching only 20 mm in length. *Oliva* spp. have a short spire with rounded shoulder, and range in colour from a very light cream to a deep cream/brown overlaid by a pattern of longitudinal zigzags in a darker tone [42]. These organisms are found in intertidal to deep water where they burrow through sand or mud substrates leaving a characteristic trail. Both predators and scavengers, these small animals feed on bivalves and crustaceans [43], themselves being prey to larger mollusks which drill into their body whorl leaving behind a characteristic and easily identifiable hole (examples of these perforations were found in the natural reference collection).

Natural accumulation of the *Oliva* shells (such as storm surges) at the archaeological sites are precluded by the elevation of Jerimalai and Lene Hara above the shore, while MK1 and MK2 are far from the coast. Similarly, while each of the four sites contain substantial economic marine shell assemblages, it is unlikely that the *Oliva* shells were gathered for subsistence given that they average around 9 mm in absolute length. Thus, while easily collected live during low-tide, one would have to collect an enormous quantity to gain enough calories to make their collection and processing worthwhile. In this way, the *Oliva* spp. shells are similar to the *Nassarius globosus* and *Nassarius pullus* shells found in levels dated post 6,500 cal. BP at these same sites [22], as well as the *Nassarius kraussianus* shell beads recovered from the Middle Stone Age levels of Blombos Cave in South Africa [4].

Instead, the *Oliva* appear to have been gathered as ornamental shell, that is, they were used exclusively for ornament-making (32). Most appear to have been collected live or shortly after death as very few exhibit the muted shell sculpture, pitting, and low intensity but widely distributed damage to their surfaces indicative of having rested on the shore for an extended period of time. This preference for undamaged shells which retained their natural shiny surface is reflected throughout the collection, with no changes in this preference identified either between the sites or through the stratigraphy.

## Results

In total, 485 *Oliva* spp. shell beads were identified and examined from Jerimalai, Lene Hara, MK1, and MK2 (Tables 1–6). These artefacts appear in the earliest layers of human occupation, in Jerimalai SQ A Spit 44 (Spit 46 dating to 43,381–41,616 cal. BP [38255±596 Wk 17831]) and Jerimalai SQ B Spit 56 (*Oliva* bead directly dated to 38,246–36,136 cal. BP) and continue throughout the stratigraphy to the most recent levels (in Jerimalai SQ B 273–184 cal. BP [124±32 Wk 19228]). An increase in their deposition occurred at 5,000–6,000 cal. BP, is evident in Jerimalai and Lene Hara, and perhaps also MK1.

Transformation of the raw *Oliva* shell into a bead required minimal modification involving only the removal of the apex so that a string could be passed through its internal structure. While a small number of the shells present evidence for having undergone beach wash, and consequently, likely exhibited a small perforation at the tip when collected, most show no evidence for having accrued natural damage prior to collection. As such, the vast majority of artefacts must have had their apex intentionally removed for use.

As was mentioned above, three approaches to removing the shell apex were experimented with in order to identify which method/s were used by the ancient Timorese people: (1) grinding on a sandstone slab; (2) percussion flaking, where the shell is held stationary and a stone is brought down against the apex; and (3) tapping, where the shell apex is forcibly tapped repeatedly on a hard surface. Traces left from each of these approaches were found to be distinctive and distinguishable from one another, as well as from naturally (beach washed) perforated shells. Shells ground on the sandstone slab exhibited a clean horizontal surface covered with parallel striations indicating the direction of grinding (Fig 3D–3F), while removal of the apex via percussion flaking was found to be difficult as the shell often slipped out of ones grasp. It also took several minutes longer to create a small perforation, and resulted in the shell apex appearing ‘battered’—having localised crushing of the shell surface with a number of striations running from the apex rim down the shell sides, this damaged caused when the stone missed the shell tip or slipped during a strike (Fig 3G–3I). Finally, the tapping-technique—where the shell was simply held in the hand and repeatedly tapped with force against the sandstone slab—took less than a minute with little effort from the actor to produce a hole large enough to pass a string through (c. 4 mm wide). This result occurred on shells that were both live collected as well as those collected post-mortem and which already exhibited a small natural hole in their apex. In these latter cases, the presence of the natural perforation accelerated the production of a hole large enough for use. Perforations created using this method were characterised by microchips around the circumference of the hole, the chips both falling away and into the shell interior (Fig 3J–3L).

The tapping-technique, was the only method of apex removal identified in the Timorese collection, including on the oldest bead recovered (that directly dated to 35,000 cal. BP from Jerimalai). Given the simplicity and ease of this method, something that could be done by young and old alike, it should not be surprising that it had such longevity.

Importantly, it was observed during experimentation that while traces of manufacture are initially clear to the observer, once the newly made bead is strung and used for even just a few hours, these traces become smoothed resulting in a perforation wall which is very similar at low magnification in appearance to a natural, beach worn opening (Fig 4E). Despite this fact, however, we were able to determine that the archaeological Timorese *Oliva* were manufactured—having had their apex deliberately removed using the tapping-technique—as, while superficially similar in appearance to beach worn shells, the apical holes in such shells only become large enough to pass a string through after having suffered significant damage from beach wash, resulting in additional holes appearing in the body whorl and an overall weakening of the entire shell. Thus, ‘naturally occurring’ *Oliva* beads are not robust enough to endure the intense use indicated by the use wear outlined below. Furthermore, not one of the archaeological examples exhibit evidence for having suffered the intensity of damage observed on the modern, beach worn specimens.

**Fig 5:**
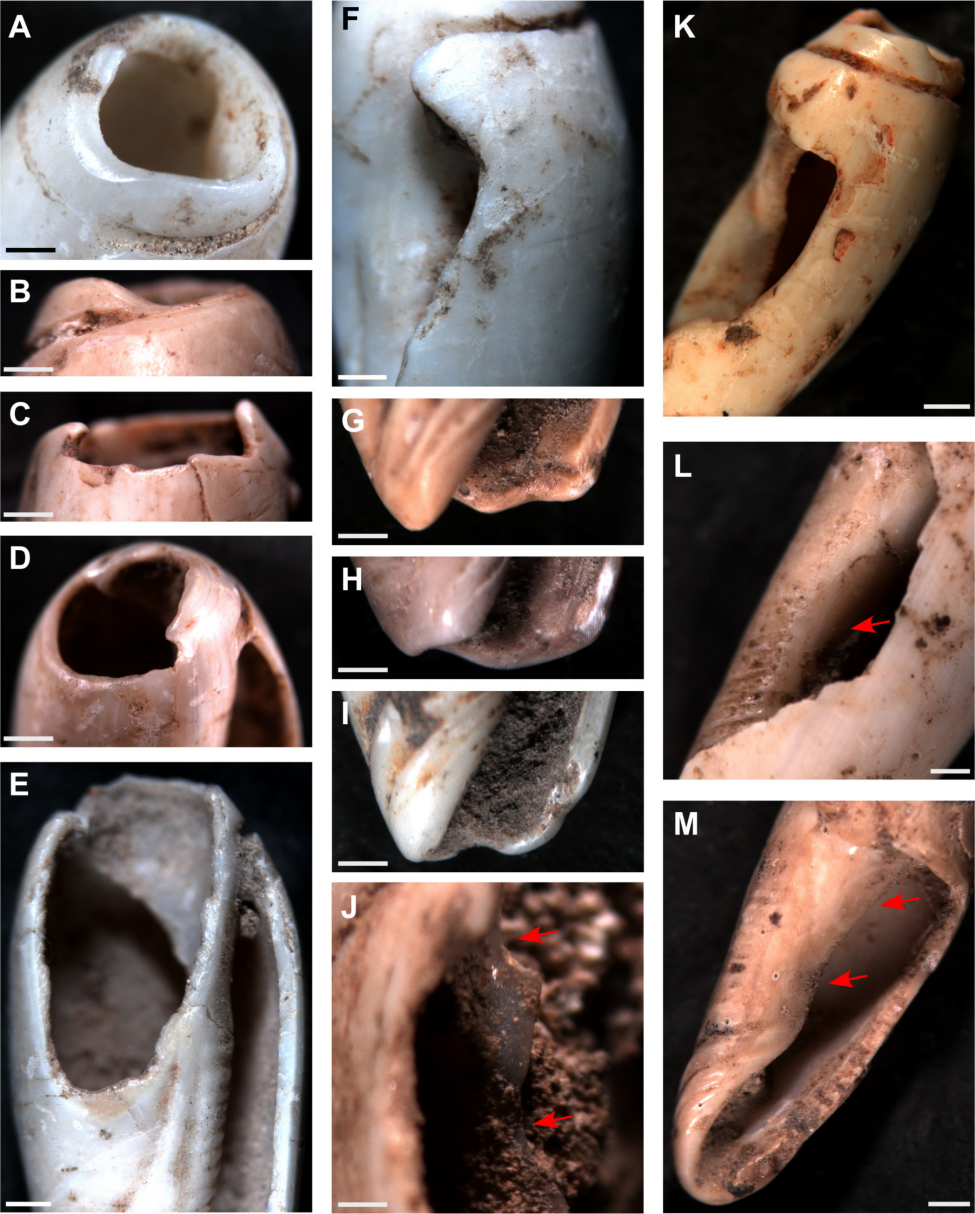
Typical use wear observed on the Timorese *Oliva* spp. shell beads. (A—E) Smoothing of the apex aperture; (F and K) notch worn into the outer lip; (G—I) smoothing and faceting of the canal; (J) notches worn into the interior structure; (L—M) facets worn into the inner lip. Bar scale = 1 millimetre.

**Fig 6:**
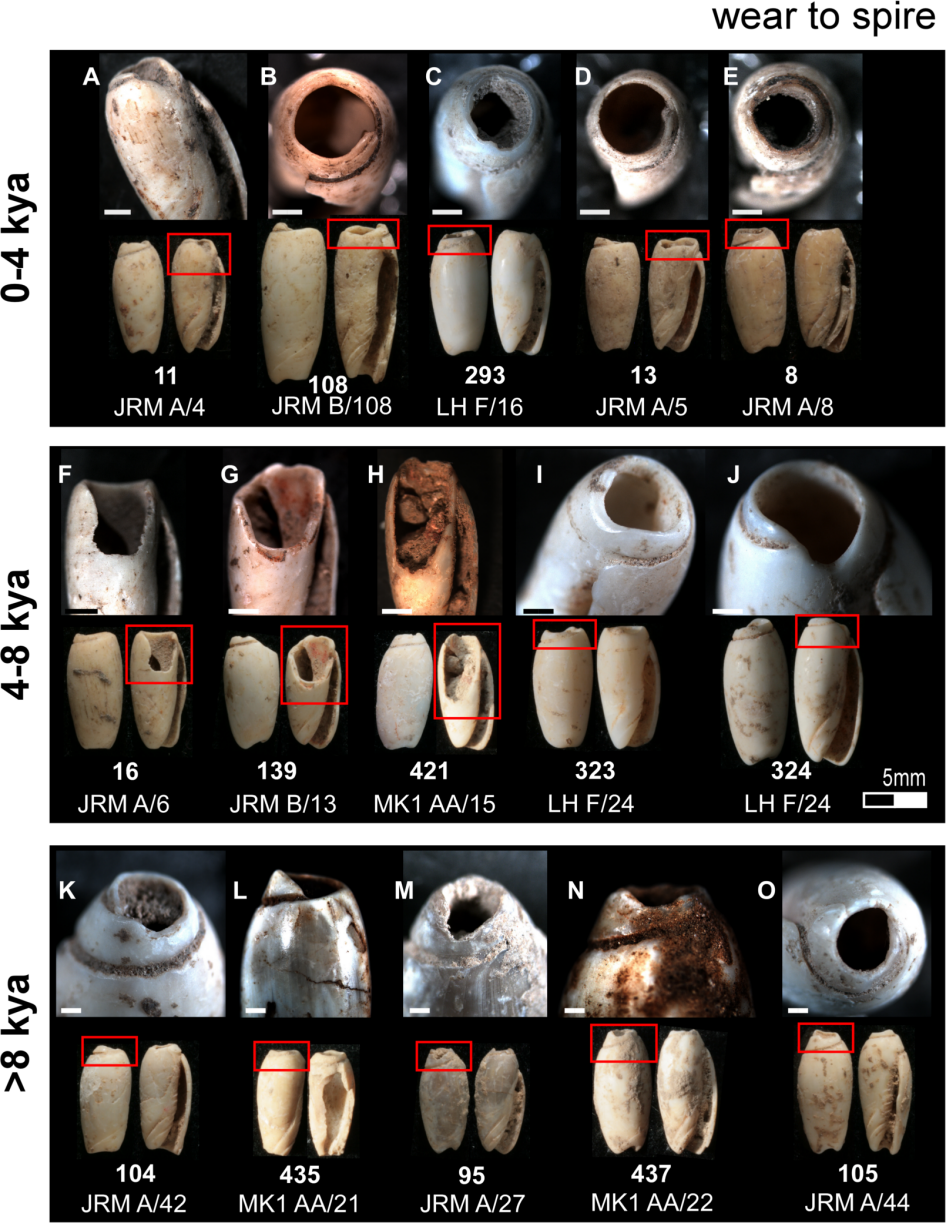
Apical perforation wear typical of beads recovered for each of the three temporal analytical units. Bar scale = 1 millimetre.

**Fig 7:**
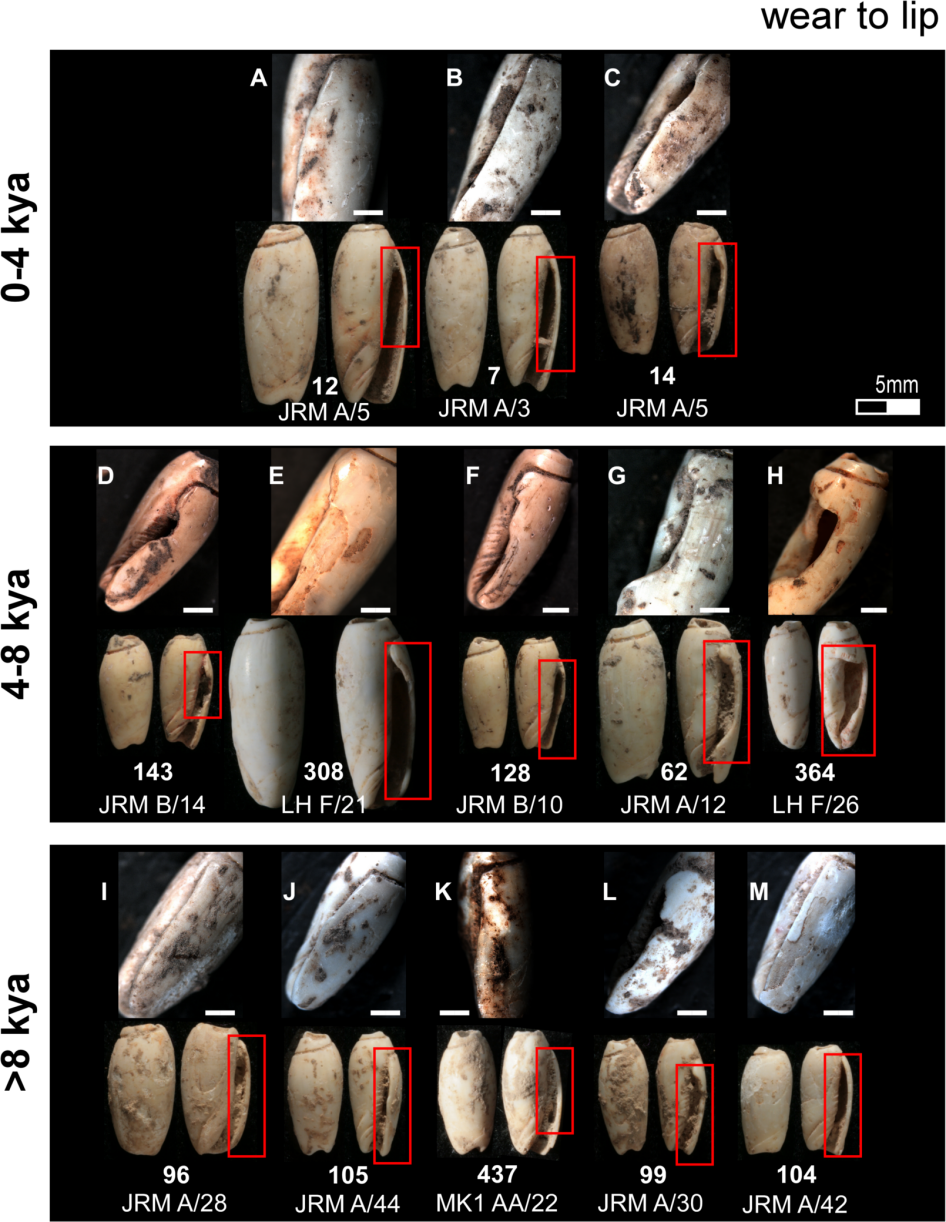
Lip use wear typical of beads recovered from each of the three temporal analytical units. Bar scale = 1 millimetre.

**Fig 8:**
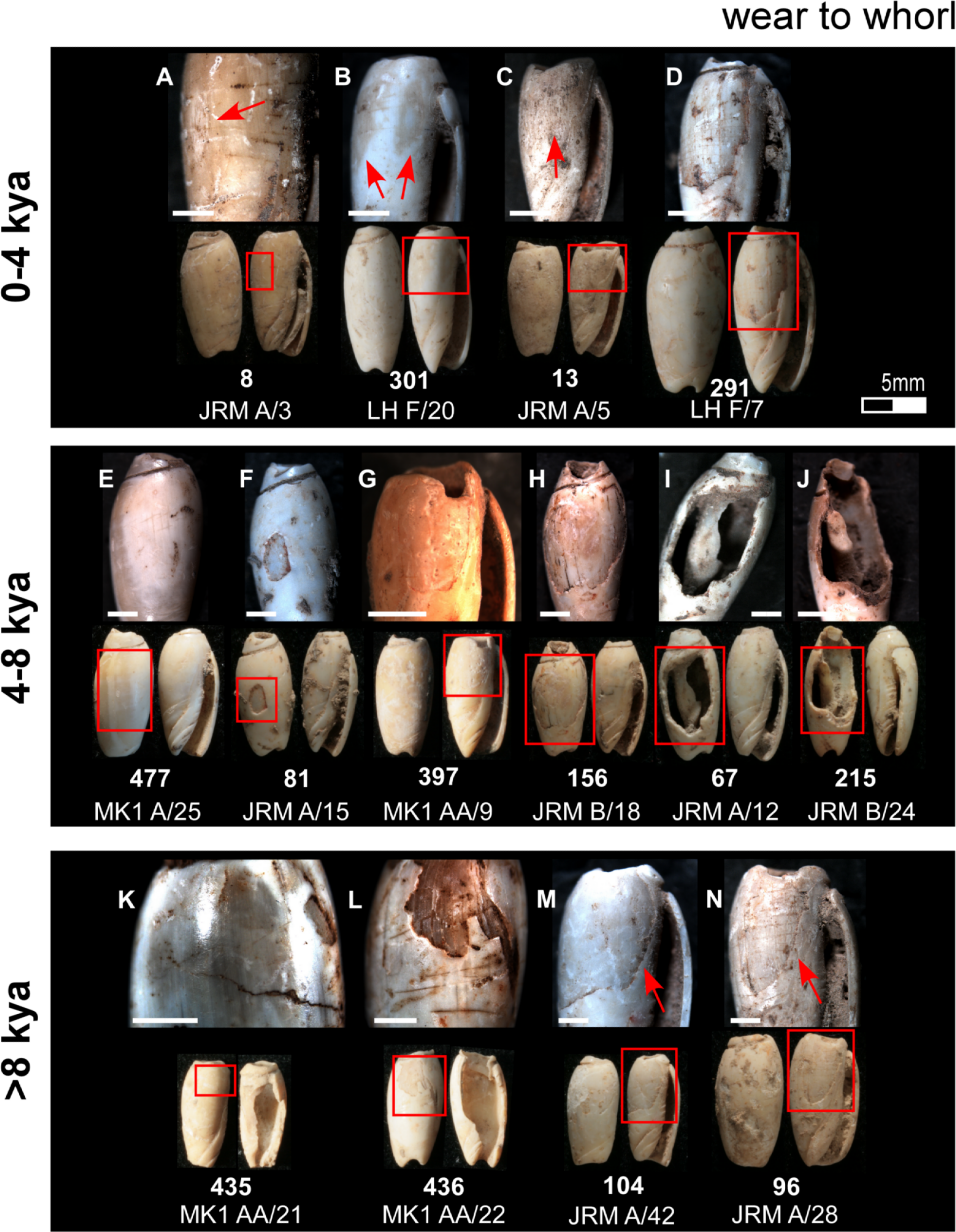
Body whorl use wear typical of beads recovered from each of the three temporal analytical units. Bar scale = 1 millimetre.

**Fig 9:**
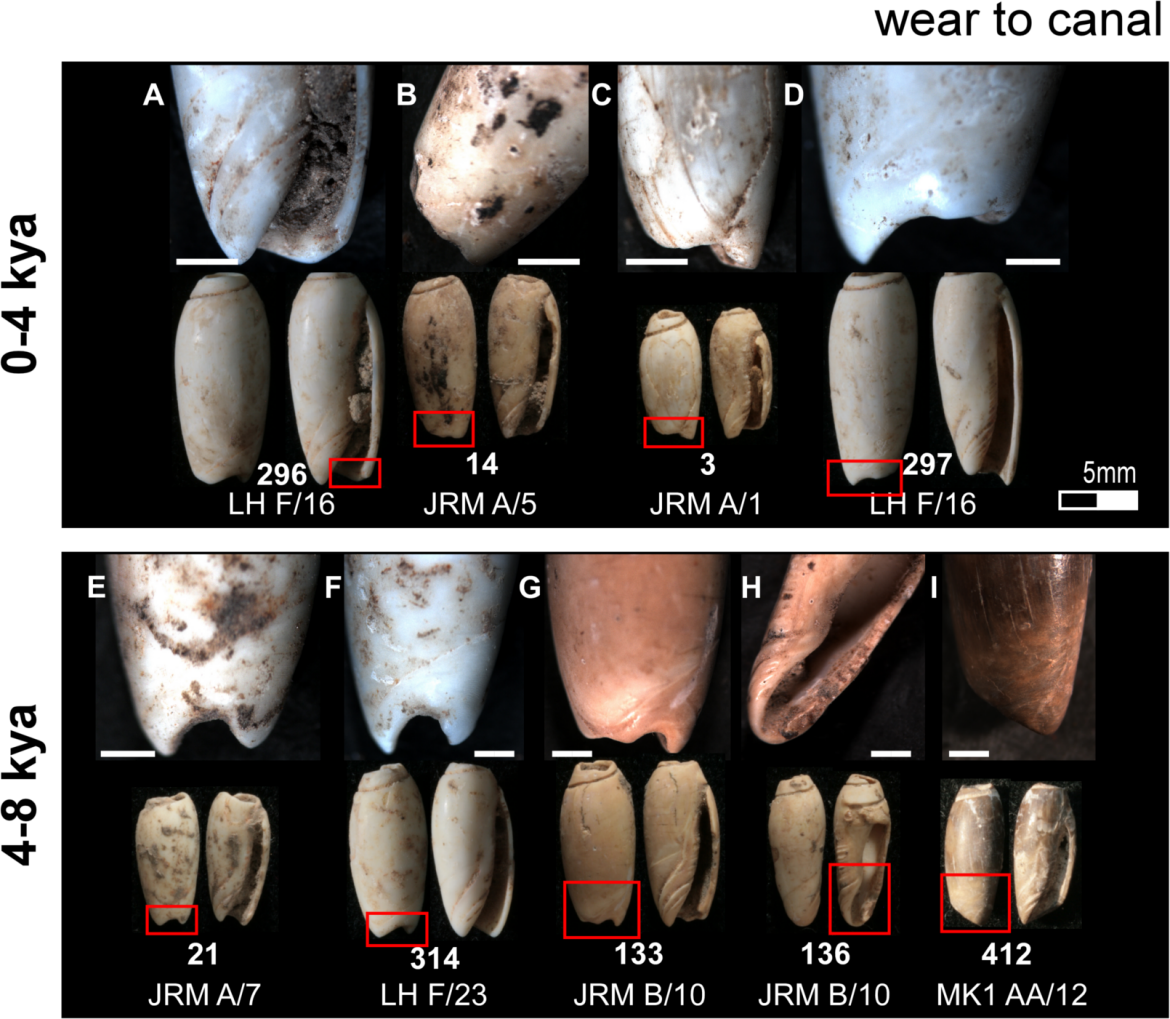
Wear to the canal typical of beads recovered from each of the three temporal analytical units. Bar scale = 1 millimetre.

Having identified the mode of perforation, the proceeding experiment attempted to replicate the use wear observed on the archaeological beads: rounding of the apex perforation edges (Fig 5A–5D), rounding and faceting of the outer lip (Fig 5F and 5K), faceting of the inner lip (Fig 5L and 5M), and rounding and faceting of the canal (Fig 5G–5I). On each of the Timorese beads, wear smooths the edges of the apical opening (Fig 5A–5D), with this opening reaching well down into the body whorl on several of the artefacts (Fig 5E and Fig 6F–6H). On numerous shells, key-holing—a small notch worn into one wall of the aperture—is evident (Fig 6A, 6B, 6J, 6M and 6O). The mesial section of the shells (whorl, inner lip, outer lip) displays the most intensive wear. Instead of being gently curved, as is the natural state of the an *Oliva* shell outer lip, the Timorese beads exhibit faceting of either isolated sections of the lip extremities, or notches ranging in width from a couple of millimetres to covering half of the lip length (Fig 7). On those featuring the most developed notching of the outer lip, faceting of the internal structure or inner lip was also recorded (Fig 5K–5M). Areas worn away through repeatedly rubbing against a surface are also a common feature on the Timorese beads. This wear type ranges between a slight wearing of the upper shell surface marked by discolouration (Fig 8A, 8B and 8E), to whole shell layers having been abraded away over small (Fig 8F and 8L), and more extensive (Fig 8D, 8G, 8H and 8L–8N) areas. Several shells are also completely worn through on their dorsal surface (Fig 8I and 8J). Observed less frequently was smoothing and faceting of the canal (Fig 9, Fig 5G–5I). Here wear ranged between small facets located on the ventral surface (Fig 9A), to lengthening of the scooped canal structure (Figs 9E and 5F), to a reduction of the anterior edge completely (Fig 9B–9D and 9G–9I).

Results of the stringing experiment found that those beads strung consecutively (as in a necklace) were accumulating wear in the same locations (apex perforation, lip, and canal) and of the same type (smoothing, faceting, key-holing) observed on the archaeological examples. Additionally, those attached to a piece of cotton canvas (Fig 4C) were beginning to show signs of wear on the higher points of their body whorl (Fig 4J), similar to that observed on many of the Timorese beads, suggesting that the archaeological beads built up this type of wear from rubbing against another surface such as skin or clothing. As can be seen in Fig 4A, when *Oliva* shells are strung consecutively, those located at the lowermost point of a necklace (or another long strand of beads) hang loosely at a 15 to 60 degree angle to the string owing to the height of the collumela. Beads located further up the string, on the other hand, will sit vertically with the sting passing through the canal. These differences in the placement of beads on a single, long strand will result in a number of the beads displaying heavier wear on the posterior sections of their columella, while on others wear is more pronounced around the canal. Such is the pattern observed in the Timorese collection. Consequently, this evidence suggests that the Timorese beads were strung as a beaded strand which was worn against the body or an item of material culture.

During experiments, several important observations were made which further elucidate how the archaeological specimens were strung—most interesting being that a sharp point was found to be necessary to string the beads in experimentation, as it was impossible to force the string through the tiny aperture without a point pushing it further into the shell structure. For the experimental beads, a metal needle point was used, and it is possible that the sharp point of a fish spine or bamboo splinter may have been similarly utilised for the archaeological examples. Fish bone has been found at each of these sites and is currently undergoing analysis.

**Fig 10:**
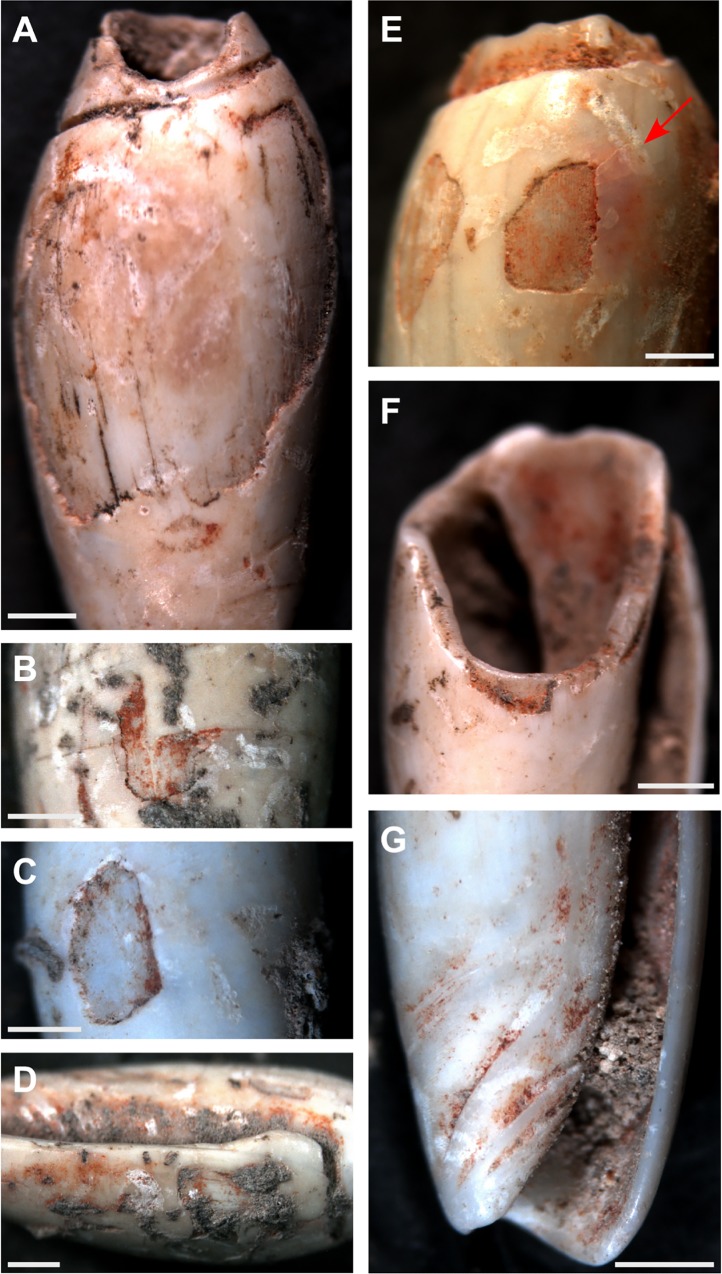
Red ochre observed on the Timorese *Oliva* spp. beads. (A—C) wear to the body whorl with (E) showing ochre working in under one of the shell layers; (D) inner lip; (F) inner shell and perforation rim; (G) fasciole. Bar scale = 1 millimetre.

The presence of a red colourant is another feature of the bead assemblages. The identification of this residue as a hematite rich pigment was determined through subjecting two of the Jerimalai *Oliva* shell beads to LA-ICP-MS analysis which recorded a significant presence of iron in its composition. Red ochre was commonly observed caught within the sutures and internal structure of the shell beads from each of the four sites (Fig 10). Concentrations were located along the inner lip (Fig 10D) and fasciole (Fig 10G), with sparser distributions around the posterior perforation and the edges of worn areas located in the body whorl (Fig 10A–10C). In several cases, ochre has worked its way under the upper layer of the shell (Fig 10E), suggesting that the bead was rubbed against an ochred surface. This distribution of colourant (gathering in creases and inner areas and absent from higher areas) suggests that it was collected passively by the beads in the process of repeated rubbing against an ochred surface, rather than the shells themselves being painted with ochre.

## Discussion

Previously, the oldest examples of marine shell beads came from poorly dated Holocene contexts throughout the region (e.g., [44]). Worked *Oliva* shells are reported for sites in Timor-Leste by Glover [45], and consist of two examples from Bui Ceri Uato, four from Uai Bobo 2, and another four from Uai Bobo 1 (Fig 1). These artefacts are described as having been “pierced by removing the apex” ([45]: 119), and from the provided plates (117: Plate 32; 152: Plate 38; 184: Plate 45), appear to display the same suite of manufacture and use traces observed on the beads presented here (flaked apex and lip damage featuring faceting). Similarly, two *Oliva* sp. shells “with the apices removed” [41] were identified from deposits dated to between c. 2,000–500 years BP in the Niah Caves, along with a single specimen found associated with red pigment from similarly aged deposits at Lobang Hangus, both in Sarawak [46]. A photograph of this last artefact from Lobang Hangus ([46]: 305) shows rounding and smoothing of the perforation less extensive than that observed on the newly recovered Timorese beads.

The evidence recovered from Jerimalai, Lene Hara, MK1, and MK2 indicate that *Oliva* shell bead production in Timor-Leste begun in the first few thousand years after initial settlement, though apparently after the selection of *Nautilus* shell for ornamental purposes [23]. At this time of early occupation, relative sea-level for the coast near to Jerimalai would have been 55 m below present [47, 48], and thus, based on the General Bathymetric Chart of the Oceans (GEBCO_14) dataset for coastline reconstruction [49], the shortest walking distance from the site to the coast some 42,000 years ago would have been approximately 2.8 km. This distance was only increased to about 3.5 km during the Last Glacial Maximum (LGM), and thus, distance to shoreline was never an issue for those visiting Jerimalai and Lene Hara for collecting marine raw materials, though it would have been more difficult to access owing to a steeper descent during the LGM, perhaps explaining why these two sites were little used during this period. For the inland sites of MK1 and MK2, the opposite was the case throughout their occupation history, and thus, it is not only interesting that these two sites contained marine materials throughout their stratigraphy, but also that the deposition rates for *Oliva* shell beads largely mirrors those of the more coastal Jerimalai and Lene Hara shelters.

Deposition of *Oliva* shell beads remains low and constant from their initial appearance in the archaeological record until levels dated to between 8,000 and 4,000 cal. BP where a sharp increase in bead numbers is found in Jerimalai and Lene Hara, with a similar pattern perhaps indicated at MK1. This increase in bead numbers ties in with a significant increase in net deposition rates for deposit sequence accumulation and quantity of shell deposited at all four sites from about 7,000 cal. BP. After around 4,000 cal. BP, frequency of *Oliva* shell beads diminishes back to a level similar to that observed for levels deposited prior to 8,000 years BP. Interestingly, the use wear observed on individual beads coming from these three analytical temporal periods (0 - 4kya; 4 - 8kya; >8kya) also reflects changes in use intensity. As shown in Figs 6–9, wear is most severe on those beads dating to between 4,000 to 8,000 cal. BP, especially around their apical perforations and body whorl. In fact, beads which displayed significant reduction of their body whorl were only found in this middle period, and as both the wear locations and types do not change, it does not appear that *Oliva* shells shifted to a different type of use, but rather that they were simply worn far more intensively during this time than both before and after this period.

This phase of increased *Oliva* shell bead usage coincides with the initial appearance of *Nassarius* shell appliqués at these same sites [22]. These two shell bead types—*Oliva* and *Nassarius*—while having been used in different ways, both indicate the increased use of marine shell for ornamentation at the time of sea-level stabilisation, and while it is tempting to simply correlate the two phenomena, the fact that it is not simply a case of *more* beads being deposited, but that each bead saw more intensive use individually argues against a simple case that such shells became more abundant or easier to collect from 8,000 cal. BP. Instead, it would appear that sea-level stabilisation and the associated increased productivity of the near shore coastal environment may have precipitated changes in settlement patterns and social interaction around Jerimalai and Lene Hara, resulting in increased use of these shelters by more people at any one time. With populations expanding in the area, it may have been that shell beads not only saw an increase in demand (to decorate more items/people) resulting in beads being used for longer, but also saw increased significance in mediating tensions brought about by a growing society—effecting increased use of the beads. After ~4000 cal. BP evidence for occupation at the sites declines dramatically and likely reflects the movement of everyday habitation out into open villages [45]. Beads dating to this period display use wear far less intensive than those dating to the period of sea-level stabilisation, and may suggest that the importance or desirability of *Oliva* shell beads had returned to a level similar to that previous to the major environmental change. Continuing analysis of both the artefactual and faunal assemblages recovered from these four sites will enable us to further elucidate the nature of these social and organisational changes in the future.

As the tempo and mode of the emergence of personal ornamentation in the first instance, and its diversification throughout the Late Pleistocene/Holocene period in different locations is considered key for understanding changes in our ancestors’ social and cultural development, the information provided by the *Oliva* beads from Timor is invaluable. In particular, they demonstrate the potential abundance of such small pieces of symbolic material culture in ISEA when modern standards of archaeological recovery are pursued. Indeed, with new excavations being undertaken across the Southeast Asian region at this time, we expect to see a complete overturning of the notion that this region was somewhat of a cultural backwater in terms of symbolic production in coming years.

## Conclusion

Analysis of more than 450 *Oliva* shell beads recovered from four archaeological sites located at the eastern end of Timor-Leste has allowed us to identify an important example of early marine shell bead use dating back to around 37,000 years ago. Microscopic analysis of the archaeological bead collection in combination with replicative experiments on modern *Oliva* shells indicates that the Timorese beads were strung consecutively creating a beaded strand which was worn against the body or an item of material culture coloured with red ochre.

While commonly reported for both Africa and Eurasia, examples of symbolic material culture in Southeast Asia are exceedingly rare, though immensely important for exploring the development of Modern Human cultural diversity in this region. An increase in bead production between ca. 8,000 and 4,000 years ago indicates that Timorese communities appear to have been undergoing significant changes in settlement and social interaction, likely in response to the changing coastal environment. One of the most significant findings of this research, however, is the demonstration of the potential richness of symbolic material culture items in early Southeast Asian sites—in direct contrast to previous perceptions.
